# Sociodemographic Profile, Health Conditions, and Burden of Informal Caregivers of Older Adults in Brazil During the COVID-19 Pandemic: Cross-Sectional, Exploratory, Noninterventional, Descriptive Study

**DOI:** 10.2196/47510

**Published:** 2023-11-23

**Authors:** Pâmela Moraes Ferreira, Mateus Cunha Gomes, Lucianne Nascimento de Araujo, Tainá Sayuri Onuma de Oliveira, Glenda Ferreira, Cintia Aben-Athar, Silvio Eder Dias da Silva, Aline M P Cruz Ramos, Diego Pereira Rodrigues, Fabianne Sousa

**Affiliations:** 1 Federal University of Para Belem Brazil

**Keywords:** informal caregivers, older adult, caregiver, caregivers, caregiving, burden, informal care, cross-sectional, gerontology, older adults, hospitalized, overload, burnout

## Abstract

**Background:**

Demographic changes in the world population have resulted in an increasingly aging society, with a progressive increase in the number of people in situations of dependence, who require assistance from family members to meet their basic needs. Caring for older adults involves performing diverse activities, resulting in reduced free time and tiredness, and fulfilling the demands and expectations related to personal, family, physical, and social life, consequently compromising the quality of life of the caregiver. In this context, the informal caregiver of hospitalized older adults emerges as the focus of attention.

**Objective:**

The aim of this study was to describe the sociodemographic profile, health conditions, and burden of informal caregivers of older adults admitted to a university hospital in Brazil during the COVID-19 pandemic period.

**Methods:**

This is a cross-sectional, descriptive, and analytical study that was conducted with 25 informal caregivers of hospitalized older adults in a university hospital in Brazil between August and September 2022. Three instruments were applied: Caregiver Burden Inventory, sociodemographic questionnaire, and health conditions questionnaire. The data were analyzed using SPSS version 28.0. Descriptive (frequency and percentage) and inferential analyses were performed using 2-sided Student *t* test with 95% CIs.

**Results:**

Of the 25 interviewees, 18 (72%) were females, 17 (46%) were married or in a stable union, 14 (56%) completed secondary education, and 11 (44%) lived with the older adults who needed care. The average age of the participants was 44 (SD 12.8) years. Regarding their health conditions, most caregivers self-reported it as good (12/25, 48%). They provided care to their father or mother older than 70 years (14/25, 56%). The Caregiver Burden Inventory analysis showed that the caregivers were the most negatively impacted in the domains of personal life overload (mean 10.8, SD 3.46; *P*=.047) and physical overload (mean 10.6, SD 2.32; *P*=.02).

**Conclusions:**

In recent years, there has been an increase in the burden on informal caregivers of hospitalized older adults in Brazil, thereby impacting their personal and physical lives. The findings of our study show that health care professionals should be trained to promote health guidelines and actions to improve the personal and physical lives of the caregiver population in Brazil.

## Introduction

The increase in human longevity has fostered numerous challenges for governments around the world to ensure the physical, social, and legal well-being of older adults and their families. Moreover, this increase in longevity has increased the physical, emotional, and social burden of caregivers of hospitalized older adults [1]. Technologies in health care are advancing, thereby proportionally increasing the longevity of the aging population. Concurrently, the challenges in caring for older adults are also increasing, and these challenges become tougher during a pandemic [1,2].

The COVID-19 pandemic affected millions worldwide, causing countless deaths; 656,864,989 death cases of COVID-19 were confirmed worldwide by the end of epidemiological week 51 of 2022 on December 24, 2022 [3]. COVID-19 infection mostly affects the upper airways; complicated cases of COVID-19 include bilateral interstitial neuropathy, severe acute respiratory failure, and organ failure, wherein ventilatory support may be required [4]. The older adult population is the most vulnerable to diseases since the natural aging process includes changes at the cellular level of tissues and organs that favor morbidity, frailty, as well as mortality [5]. In addition, the aging immune system is intrinsically related to the severity of the disease. Currently, the prevalence of sequelae of COVID-19 still exists called the long COVID, which represents an emerging global crisis. The quantifiable risks of COVID-19 and their biological associations are poorly resolved [5,6].

The family is assigned the responsibility of assisting its aging members. Family caregivers are part of the informal support network that consists of family members, friends, acquaintances, and neighbors who work without remuneration and are called informal caregivers. This fact marks the difference from the formal network of caregivers composed of health care professionals, whether at home, hospitals, or outpatient institutions [2-6]. Informal care is the most frequent model of care for older adults. Many relatives prefer informal care from their family members, possibly because of their cultural values, lack of adequate formal care services, or the lack of financial resources to hire a formal caregiver. The family is considered responsible for meeting the social and health demands of their older adults, thus necessitating a qualified and constant support system [6,7]. In light of this, the informal caregiver is responsible for the care of older adults who are vulnerable to infections and complications and thus require more friendly care, which can generate a high burden on the responsible caregiver, triggering challenging questions regarding the function and health of the caregiver [7]. Studies [8-11] have shown that the caregivers’ sociodemographic profiles are one of the main factors contributing to their burden. Caregivers experience stress, illness, and emotional burdens, thereby affecting their ability to provide adequate care to older adults. Stress has been reported in 81.8% of the interviewed caregivers [10]. In addition, lack of support was reported by caregivers with moderate and severe anxiety and depression in a survey [11], leading to overload.

Overload is specifically defined as a resistance to the provision of care, caused by the inclusion or expansion of activities performed in care. Overload comprises 2 dimensions: objective and subjective. Objective overload is related to the activities carried out in the provision and supervision of care as well as to the disturbances and limitations imposed on the social and professional life of the caregiver and the financial upheavals. Subjective overload refers to the understanding and affections of the caregivers, apprehensions about the patient, feeling of carrying a weight, and discomfort in the exercise of care [11].

In the context of support for caregivers, the Brazilian reality differs from that of the other member countries of the Organization for Economic Cooperation and Development, which is constituted as an international organization composed of advanced and emerging countries that have policies to support the family caregivers of older adults, social responses and support services, financial benefits, and work flexibility (time reduction, flexible hours, teleworking, etc) [12-14]. The informal demand for health care provision by caregivers drives the institutionalization of older adults, as the caregivers cannot bear the burden. The main reasons for institutionalization listed in a study [15] were the presence of family issues, abandonment, and violence. Another study [16] listed dependency in activities of daily living and the symptomatology of depression as causes, which caused the loss of independence at home, thereby generating the need for specific care, to which the informal care network did not guarantee a response.

Our study is important because this is the first study to use the Caregiver Burden Inventory instrument with informal caregivers to assess their burden in a hospital environment in the Amazon region of South America. Our study not only informs about the burden of informal caregivers of hospitalized older adults in the Amazon region, with a view of contributing to public policies aimed at this group, but also provides a basis for the development of innovative and specific strategies aimed at supporting informal caregivers who have excessive burden while caring for older adults. From this perspective, our study aims to describe the sociodemographic profile, health conditions, and burdens of informal caregivers of older adults admitted to a university hospital in Brazil during the COVID-19 pandemic period.

## Methods

### Study Design

This is a cross-sectional, exploratory, noninterventional, descriptive, and analytical study. The STROBE (Strengthening the Reporting of Observational Studies in Epidemiology) checklist [10] for observational studies was used for conducting this research and reporting the results obtained.

### Location, Study Population, Sample, and Period

Data collection was performed during the preoperative or postoperative period (up to 24 hours after surgery or up to 7 days after a surgery) in the surgical unit of Hospital Universitario João de Barros Barreto, with informal caregivers who accompanied older adults in clinically stable conditions, that is, without surgical complications, undergoing any surgical procedure under the supervision of a multidisciplinary team made up of doctors, nurses, dentists, psychologists, and social workers. The intentional and convenience nonrandom sampling technique was used to obtain the sample population. The health team at the inpatient unit was asked to identify all the informal caregivers who accompanied older adults in the inpatient unit to perform the interviews, considering the restrictions on in-person care in the hospital complex during the pandemic period. Data were collected between August and September 2022. The instruments were applied by the researcher in a specific room in order to guarantee the confidentiality of the data, with an average duration of 20 minutes, as it was still the pandemic period, following World Health Organization’s recommendations regarding the use of masks, minimum social distancing of 1 meter, and hand hygiene.

### Inclusion and Exclusion Criteria

In Brazil, the role of an informal caregiver is defined by the person who, in the private domestic space, performs or helps a person with limitations to perform their basic and instrumental activities of daily living, with the objective of preserving their autonomy and independence. These activities range from personal hygiene to family financial management. The main or primary caregiver assumes all or most of the responsibility for caring and is the one who performs most of the activities. Secondary and tertiary caregivers are those family members, friends, neighbors, and volunteers who complement the help, generally providing less support [8].

The following inclusion criteria were adopted.

1. For informal caregivers: aged 18 years or older; be a primary, secondary, and tertiary informal caregiver; not being paid for the care provided to older adults; ability to answer the instrument’s questions; and have time to respond to the research instruments.
2. For older adults: aged 60 years and older; hospitalized in the surgical inpatient unit for any surgical or preoperative procedure; and be clinically stable.

The exclusion criteria were as follows.

1. Formal caregiver and caregivers who were unable to respond to the instruments for some reason (difficulty understanding the questions and having cognitive impairment or being unable to complete the answers to the instruments).
2. Older adults: accompanied by a formal caregiver; alone at the time of the interview; and present any change in their clinical condition.

### Study Instruments

#### Sociodemographic Questionnaire and Health Conditions Questionnaire

Two instruments were applied. The sociodemographic questionnaire consisted of sociodemographic questions for the informal caregiver, such as age, sex, marital status, education, whether the caregiver lived with the older adult, health status, whether this person was affected by the provision of care, whether the caregiver was responsible for the care of another older adult, and degree of kinship. The health conditions questionnaire assesses the health conditions of the caregivers and the age and sex of the older adults being cared for.

#### Caregiver Burden Inventory

The burden of care provided by informal caregivers was assessed using a version of the Caregiver Burden Inventory instrument, which was adapted and validated in Portuguese. This inventory contains 24 questions divided into 5 domains. The first to fifth deal with the time-dependent load, personal life load, physical load, social load, and the emotional load, respectively. Each question had the options “completely disagree,” which is equivalent to 0 points, “disagree” corresponding to 1 point, “neither agree nor disagree” corresponding to 2 points, “agree” corresponding to 3 points, and “completely agree,” which corresponded with 4 points in the analysis load. The results were interpreted by calculating the scores separately, except for the physical overload domain, which was multiplied by 1.25, as it had only 4 items, and this multiplication was done if it was necessary to make comparisons between the domains. After the calculation, it was possible to identify the score present in each domain. The total score could be obtained by adding the domains. The instrument’s psychometric properties were similar to those obtained by the original Canadian instrument, whose Cronbach α values were between .73 and .86 [17].

### Statistical Analysis

The data were organized in the Microsoft Office Excel program. Subsequently, the SPSS (version 28; IBM Corp) program was used for descriptive analysis (frequency and percentage) and inferential analysis, applying the parametric 2-sided *t* test, which was chosen due to the small sample size; 95% CI was used with a significance level of *P*<.05.

### Ethics Approval and Consent

This study follows the standards of the 2013 Declaration of Helsinki, ensuring confidentiality and anonymity of data of all participants, with approval by the ethics committee on Human Research of the Federal University of Pará in accordance with Resolution 466/2012 of the National Health Council (CNS in Portuguese; approval 5.312.450). All interviewees who agreed to participate in the research were presented with the objectives, risks, and benefits of the study, and after providing the information, they signed the free and informed consent form, received a copy of it, and proceeded with the interview.

## Results

The sample population in this study consisted of 25 informal caregivers of older adults hospitalized in the surgical inpatient unit of the Hospital Universitario João de Barros Barreto in Belém, Pará, Brazil. The interviewees did not have any link and were not members of any caregiver organization. The mean age of the caregivers was 44 (SD 12.8) years; most caregivers were females (18/25, 72%), married (17/25, 46%), completed high school (14/25, 56%), and were living in the same house as the older adult (11/25, 44%) (Table 1).

**Table 1:** Demographic profiles of informal caregivers of older adults hospitalized in the surgical inpatient unit of the university hospital in Belém, Pará, Brazil in 2022 (N=25).

Variables		Values, n (%)
Not a member of an organization for informal caregivers		25 (100)
**Age (years)**		
	20-29	4 (16)
	30-39	5 (20)
	40-49	7 (28)
	50-59	5 (20)
	≥60	4 (16)
**Gender**		
	Female	18 (72)
	Male	7 (28)
**Marital status**		
	Married or stable union	17 (46)
	Single	6 (32)
	Widower	2 (22)
**Education**		
	Illiterate	1 (4)
	Incomplete elementary school (grades 1-4)	1 (4)
	Completed elementary school (grades 5-9)	3 (12)
	High school (grades 10-12)	14 (56)
	Incomplete higher education	1 (4)
	Completed higher education	5 (20)
**Where do you live in relation to the person for whom you are the primary caregiver?**		
	In the same house	11 (44)
	In different houses but in the same building	4 (16)
	They are neighbors and live on the same street or nearby	7 (28)
	Live less than 30 minutes away (one way)	2 (8)
	Lives between 30 minutes and 1 hour of travel (one way)	1 (4)

Table 2 shows that informal caregivers who accompanied the older adults hospitalized in the surgical unit rated their health status as good (12/25, 48%) and cared for their father or mother (14/25, 56%) aged 70 years and older (14/25, 56%) and that most older adults were females (14/25, 56%).

**Table 2:** Health condition profiles of the informal caregivers accompanying the hospitalized older adults in the surgical inpatient unit of the university hospital in Belém, Pará, Brazil in 2022 (N=25).

				Values, n (%)
**Informal caregiver**				
	**How do you rate your health condition?**			
		Bad	1 (4)	
		Neither good nor bad	10 (40)	
		Good	12 (48)	
		Very good	2 (8)	
	**What is your relationship to the person for whom you are the primary caregiver, that is, to whom you provide most of your care?**			
		Father or mother	14 (56)	
		Father-in-law	2 (8)	
		Spouse or partner	4 (16)	
		Brother-in-law or sister-in-law	5 (20)	
**Older adult**				
	**Age group (years)**			
		60-69	11 (44)	
		≥70	14 (56)	
	**Sex**			
		Male	11 (44)	
		Female	14 (56)	

Regarding the stratification of the Caregiver Burden Inventory scores, the greatest burden was evidenced in the domain of personal life overload (mean 10.8, SD 3.46; *P*=.047), followed by physical overload (mean 10.6, SD 2.32; *P*=.02) and emotional overload (mean 5.24, SD 2.65; *P*=.02) with a statistically significant difference in these domains (*P*<.05). The total caregiver burden score was quantified as 42.0 (SD 9.22) (Table 3).

**Table 3:** Distribution of the mean (SD) scores of the Caregiver Burden Inventory domains and total score for the sample studied in the university hospital in Belém, Pará, Brazil in 2022.

Domains	Mean (SD) score	Odds ratio (95% CI)	*P* value
Time-dependent overload	7.16 (3.03)	0.993 (0.18 to 1.04)	.99
Overload to personal life	10.8 (3.46)	1.113 (0.82 to 1.25)	.047^a^
Physical overload	10.6 (2.32)	1.138 (–0.28 to 1.42)	.02^a^
Social overload	8.56 (2.94)	0.087 (–0.13 to 0.96)	.91
Emotional overload	5.24 (2.65)	0.954 (0.42 to 1.21)	.014^a^
Total score	42.0 (9.22)	0.0765 (–0.03 to 1.09)	.42

^a^Significant at *P*<.05.

In Table 4, the items of each domain are presented. The most notable findings were that 16 (64%) respondents “expected things to be different at this time in their life” under the domain of overload to personal life, and 17 (68%) respondents agreed that they were not getting enough sleep and were physically tired under the domain of physical overload.

**Table 4:** Relative distribution of simple frequency and mean (SD) of the response options per item of the instrument in the studied sample in Belém, Pará, Brazil in 2022 (N=25).

Domain or item			Totally disagree, n (%)		I disagree, n (%)		I neither agree nor disagree, n (%)		I agree, n (%)		Totally agree, n (%)
**Time-dependent overload**											
	The person I care for	4 (16)		13 (52)		3 (12)		5 (20)		0 (0)	
	The person I care for is dependent on me	2 (8)		19 (76)		1 (4)		3 (12)		0 (0)	
	I have to be constantly aware of the person I care for	0 (0)		17 (68)		4 (16)		4 (16)		0 (0)	
	I have to help the person I care for with many basic functions (feeding, eliminations, hygiene, and getting around)	1 (4)		18 (72)		3 (12)		3 (12)		0 (0)	
	I don’t get a minute rest in my care work	1 (4)		8 (32)		11 (44)		5 (20)		0 (0)	
**Overload to personal life**											
	I feel that I am failing to live my life	2 (8)		9 (36)		7 (28)		7 (28)		0 (0)	
	I wish I could get out of this situation	2 (8)		6 (24)		6 (24)		11 (44)		0 (0)	
	My social life has been impaired	1 (4)		3 (12)		7 (28)		14 (56)		0 (0)	
	I feel emotionally exhausted from caring for this person	2 (8)		7 (28)		4 (16)		12 (48)		0 (0)	
	I had hoped that things would be different at this point in my life	0 (0)		4 (16)		5 (20)		16 (64)		0 (0)	
**Physical overload**											
	I am not getting enough sleep	0 (0)		2 (8)		6 (24)		17 (68)		0 (0)	
	My health has been impaired	2 (8)		7 (28)		10 (40)		6 (24)		0 (0)	
	Caring for this person has made me physically ill	3 (12)		6 (24)		12 (48)		4 (16)		0 (0)	
	I am physically tired	0 (0)		3 (12)		5 (20)		17 (68)		0 (0)	
**Social overload**											
	I don’t get along with other family members as well as I used to	2 (8)		9 (36)		6 (24)		8 (32)		0 (0)	
	My caregiving actions are not valued by other family members	2 (8)		11 (44)		6 (24)		6 (24)		0 (0)	
	I have had problems in my relationship with my partner	3 (12)		16 (64)		4 (16)		2 (8)		0 (0)	
	I have not been working as well as I used to (I work outside or at home)	0 (0)		6 (24)		7 (28)		12 (48)		0 (0)	
	I resent other relatives who could help but don’t	0 (0)		14 (56)		6 (24)		4 (16)		1 (4)	
**Emotional overload**											
	I feel embarrassed or uncomfortable with the behavior of the person I care for	4 (16)		17 (68)		3 (12)		1 (4)		0 (0)	
	I feel ashamed of the person I care for	4 (16)		18 (72)		3 (12)		0 (0)		0 (0)	
	I resent the person I care for	3 (12)		19 (76)		2 (8)		1 (4)		0 (0)	
	I feel uncomfortable when I receive friends	4 (16)		19 (76)		2 (8)		0 (0)		0 (0)	
	I get irritated with my interaction with the person I care for	3 (12)		17 (68)		1 (4)		4 (16)		0 (0)	

## Discussion

The Caregiver Burden Inventory is an instrument that aims to assess caregiver burden so that interventions can be designed to support caregiver activities, including the need for better support of caregivers by health care professionals. In Brazil, informal caregivers are not members of any organization for caregivers, reflecting the absence of an external direction in older adult care, unlike that for caregivers in Tondela, Portugal [18]. The burdens of the caregivers reveal that there is a need for support in public policies, as their burden reflects their vulnerability to emerging diseases [19]. In Brazil, the 1988 constitution considers the responsibility of the family, society, and the state to support older adults, in addition to providing subsidies that guarantee their participation in the community, defense of their dignity and well-being, and guarantee of their right to life [18]. In Brazil, there is still no specific law for the informal caregiver. However, in Portugal, since 2019, a statute has been created for the informal caregiver, which consists of a series of measures referring to cash benefits, tax exemptions, and forms of legal protection for male and female workers who need flexible working hours or ways of working because they are caregivers and integration of social and health services in hospitals, primary care teams, long-term care providers, and professional associations [19]. There is also a growing investment in policies for the innovative use of technologies, with the aim of improving competence and the ability to care [20].

The sociodemographic profile of the caregivers in this study shows that they were mostly females. This finding corroborates that of a study [21] carried out in Maranhão, Brazil, wherein the role of a caregiver was performed mostly by women. Culturally, in a society, the act of caring is mainly provided by women, despite several discussions about the interference of patriarchal culture in the determination of gender roles [21]. Most caregivers in this study were married. Being married enabled caregivers to better understand the needs of hospitalized older adults, with whom they could build strong and trusting relationships and thereby respect and fulfil their last wishes [22]. Most informal caregivers in this study had completed high school. Higher education is directly linked to the provision of better care. The level of education of caregivers influences their ability to learn and understand the guidelines provided by health professionals, which may possibly influence the quality of care offered to hospitalized older adults as well as the performance of the caregiver in the face of emergency situations. The greater dexterity in carrying out care activities at home, favored by education, can influence the caregiver’s perception of care overload [18].

The informal caregivers in our study were relatively young, with a predominance of young adult children responsible for older adult care. Giving care at a young age coupled with daily hours of employment increases the burden. Further, these young caregivers have higher levels of emotional discomfort and feelings of sadness when they take care of an older adult who is a close family member [21,22]. Another study [23] showed that older age was correlated with symptoms of decreased physical and emotional vitality, which were also observed in this study.

Our study shows that informal caregivers evaluated their health status as good and that they were not affected by providing care to older adults, possibly because of the greater independence of the older adults cared for, as informal caregivers participating in another survey considered their health to only be normal (neither great nor bad) as the individual being cared for had comorbidities and greater dependence [24]. In Brazil, there is a cultural value of the family taking responsibility for the care of its members. Family members who care for older adults, although are happy with this role, are subject to numerous sources of stress, arising from the task definitions of a role for which they are often not prepared as well as the repercussions on their daily lives [24,25].

The sum of the Caregiver Burden Inventory scores was higher (mean 42.0, SD 9.22) than that verified in a national study, carried out in a large urban center, which used the same scale (mean 36.4, SD 22.8) [17]. Regarding the time-dependent overload domain, a low score was evidenced in this study. This domain concerns restrictions on the caregiver’s time, that is, if he or she needs to be constantly attentive to the older adult without rest [19]. This finding is contrary to the analysis [18] that found greater overload in the time-dependent domain, possibly because that study targeted the caregivers of dependent older adults, in addition to receiving possible help from other family members, implying part-time dedication of the caregiver.

The overload on the personal life domain presented the highest score, revealing that caregivers wanted to escape their current situation. This domain analyzes the caregiver’s feelings in relation to the time spent caring for the older adults—if he or she has to stop living his or her life to take care of the older adult or if he or she would like to stop performing this caregiving function [19]. The social lives of the caregivers in this study were impaired, and they felt emotionally drained. The results of this study corroborated the findings of a scoping review [25], which showed that the informal caregivers are impacted in their daily activities. Such reports may indicate difficulty for individuals to give up their personal and professional lives to become informal caregivers in the future.

In the physical overload domain, most caregivers claimed to be physically tired. This domain analyzes aspects related to the caregiver’s health and the physical fatigue resulting from the care process [19]. A similar result was found in a study carried out at a philanthropic hospital in Paraíba, Brazil, which showed that caregivers had problems with sleep, rest, and signs of weakening of their immune system. The task of caring for older adults is usually added to other day-to-day activities, which can trigger a greater burden. Most individuals who disagreed about the presence of physical fatigue in our study were males, which may be related to the social imaginary that sees men as invulnerable, virile, and strong [26,27].

As for the social overload domain, most of the interviewees pointed out that they could not work as well as they used to. The social burden domain assesses the damage caused by care in the caregivers’ relationship with other people and in their performance in their formal employment. This domain also measures the help received from other family members to take care of the older adults [19]. Similar results were obtained with a multidimensional caregiver burden inventory scale in Canada [28]. The findings of this domain were also corroborated by those in a study carried out with informal caregivers in the city of Recife (Brazil), wherein in addition to the caregiver already being in a context of vulnerability, there were cases of job abandonment to the detriment of care. In such situations, the government must implement public policies for this specific population. In this context, in an interview with informal caregivers in Portugal, requests for subsidy from Social Security (a Portuguese government institution that provides social and financial support) were reported to take care of the dependent family member. Unlike in Portugal, there is no specific initiative in Brazil to benefit family caregivers, making this situation extremely precarious [29,30].

The lowest score was obtained for the emotional overload domain. This domain assesses the feelings of embarrassment, shame due to the person being cared for, resentment toward the person, and discomfort in the presence of other people [19]. Scale validation studies in China [31] and Brazil [19] also reported the lowest scores for emotional overload. Despite the difficulties, informal caregivers like to take care of their family members. However, a portion of the caregivers were irritated with the interaction with the person receiving care; such a relationship resulted in conflicts, mostly due to the lack of appreciation by the individual cared for. Thus, it can be considered that informal caregivers also face emotional burdens [31].

This study had limitations because there is very little research regarding care of informal caregivers for hospitalized older adults during the COVID-19 pandemic, especially in the Amazon region. Our study was the first to use the Caregiver Burden Inventory during the COVID-19 pandemic within the hospital context. Thus, our study contributes to the discussion of the role of the state in relation to the organization of a formal support network for families with hospitalized older adults and their caregivers in Amazonian municipalities in the context of the Unified Health System.

Our study shows that informal caregivers of older adults experience the greatest impact on their personal lives, followed by physical overload. The pandemic and postpandemic contexts directly and intensely affected the older adults due to immune system weakness that made them more susceptible to infection, requiring care provided by their caregivers. Health professionals who aid families with hospitalized older adults should make greater use of instruments to assess caregiver burden in their practice. Future research in the form of longitudinal studies is necessary for better investigations into factors related to overload. This study can be a valid tool for addressing the need to carry out research that directs the development of strategies that can contribute to making the daily lives of caregivers less exhausting, promoting the reduction of the burden attributed to the practice of care, and consequently, overload.

## Appendix

Multimedia Appendix 1 STROBE checklist.

## Abbreviations

STROBE: Strengthening the Reporting of Observational Studies in Epidemiology

## Data Availability

The data sets used and analyzed during this study are available from the corresponding author upon plausible justification (FS, fabiannesousa@hotmail.com).

## References

[1] Veras R, Oliveira M (2016). Care pathway for the elderly: detailing the model. Rev Bras Geriatr Gerontol.

[2] (2020). Boletim epidemiológico. Ministério da Saúde, Secretaria de Vigilância em Saúde.

[3] Su Y, Yuan D, Chen DG, Ng RH, Wang K, Choi J, Li S, Hong S, Zhang R, Xie J, Kornilov SA, Scherler K, Pavlovitch-Bedzyk AJ, Dong S, Lausted C, Lee I, Fallen S, Dai CL, Baloni P, Smith B, Duvvuri VR, Anderson KG, Li J, Yang F, Duncombe CJ, McCulloch DJ, Rostomily C, Troisch P, Zhou J, Mackay S, DeGottardi Q, May DH, Taniguchi R, Gittelman RM, Klinger M, Snyder TM, Roper R, Wojciechowska G, Murray K, Edmark R, Evans S, Jones L, Zhou Y, Rowen L, Liu R, Chour W, Algren HA, Berrington WR, Wallick JA, Cochran RA, Micikas ME, Wrin Terri, Petropoulos Christos J, Cole Hunter R, Fischer Trevan D, Wei Wei, Hoon Dave S B, Price Nathan D, Subramanian Naeha, Hill Joshua A, Hadlock Jennifer, Magis Andrew T, Ribas Antoni, Lanier Lewis L, Boyd Scott D, Bluestone Jeffrey A, Chu Helen, Hood Leroy, Gottardo Raphael, Greenberg Philip D, Davis Mark M, Goldman Jason D, Heath James R, ISB-Swedish COVID-19 Biobanking Unit (2022). Multiple early factors anticipate post-acute COVID-19 sequelae. Cell.

[4] García-Álvarez José L, García-Vigil José L (2020). Guidelines for clinical management of SARS-CoV-2 infection. Gac Med Mex.

[5] da Cruz NAO, Andriani MT, Pimenta TS, et al (2022). Repercussion of COVID-19 infection in the elderly: an integrative review. Res Soc Dev.

[6] Galdino S, Soares A, Ribeiro R, Porto M, Pereira F (2020). Impacto da covid-19 na população idosa. Plataforma Espaço Digital.

[7] Caparrol AJS, Martins G, Barbosa GC, et al (2022). Pandemia da COVID-19: quem cuida dos cuidadores informais de idosos?. Recien.

[8] Chiao C-y, Wu H-s, Hsiao C-y (2015). Caregiver burden for informal caregivers of patients with dementia: A systematic review. Int Nurs Rev.

[9] Rocha MPF, Vieira MA, Sena RRD (2008). [Unveiling the routine of informal caregivers for the elderly]. Rev Bras Enferm.

[10] Souza LRD, Hanus JS, Dela Libera LB, Silva VM, Mangilli EM, Simões PW, Ceretta LB, Tuon L (2015). Sobrecarga no cuidado, estresse e impacto na qualidade de vida de cuidadores domiciliares assistidos na atenção básica. Cad Saúde Colet.

[11] Felipe SGB, Oliveira CEDS, Silva CRDT, Mendes PN, Carvalho KMD, Lopes Silva-Júnior Fernando, Figueiredo MDLF (2020). Anxiety and depression in informal caregivers of dependent elderly people: an analytical study. Rev Bras Enferm.

[12] Pedreira LC, Oliveira AMS (2012). [Caregivers of dependent elderly at home: changes in family relationships]. Rev Bras Enferm.

[13] (2015). Brazil's development co-operation. OECD.

[14] Boas práticas internacionais e do Brasil de apoio ao cuidador familiar. Eurosocial Programa.

[15] Lopes VM, Scofield AMTS, Alcântara RKL, et al O que levou os idosos à institucionalização? / What taken the elderly people to institutionalization?. Rev Enferm UFPE.

[16] Neves HMF Causas e consequências da institucionalização de idosos : estudo tipo série de casos. @uBibliorum.

[17] Valer DB, Aires M, Fengler FL, Paskulin LMG (2015). Adaptation and validation of the Caregiver Burden Inventory for use with caregivers of elderly individuals. Rev Lat Am Enfermagem.

[18] Aires M, Fuhrmann A, Mocellin D, Pizzol F, Sponchiado L, Marchezan C, Bierhals Carla Cristiane Becker Kottwitz, Day Carolina Baltar, Santos Naiana Oliveira Dos, Paskulin Lisiane Manganelli Girardi (2020). Burden of informal caregivers of dependent elderlies in the community in small cities. Rev Gaucha Enferm.

[19] Liu Z, Heffernan C, Tan J (2020). Caregiver burden: A concept analysis. Int J Nurs Sci.

[20] Perista P (2019). A new formal status for informal carers in Portugal. A new formal status for informal carers in Portugal, ESPN Flash Report 2019/55, European Social Policy Network (ESPN), Brusselsuropean Commission (2019).

[21] Santos MAM, Coura AS, França ISX, et al (2022). Sobrecarga e qualidade de vida dos cuidadores de pessoas acamadas em domicílio. Acta paul enferm.

[22] Daely S, Nuraini T, Gayatri D, Pujasari H (2021). Impacts of age and marital status on the elderly's quality of life in an elderly social institution. J Public Health Res.

[23] Monterrosa Quintero A, Echeverri Rios AR, Fuentes-Garcia JP, Gonzalez Sanchez JC (2022). Levels of physical activity and psychological well-being in non-athletes and martial art athletes during the COVID-19 pandemic. Int J Environ Res Public Health.

[24] Rodrigo-Baños Virginia, Moral-Pairada MD, González-de Paz Luis (2021). A comprehensive assessment of informal caregivers of patients in a primary healthcare home-care program. Int J Environ Res Public Health.

[25] de GL, Oliveira M, Souza P, Campos G (2022). A autonomia e a saúde do cuidador informal: uma revisão narrativa. Revista Corpus Hippocraticum.

[26] Nascimento EMA, Rodrigues MSD, Evangelista EB, et al (2021). Estresse emocional entre cuidadores informais de pacientes em cuidados paliativos [Emotional stress between informal caregivers of patients in palliative care] [Estrés emocional entre los cuidadores informales de pacientes en cuidados paliativos]. Revista Enfermagem Uerj.

[27] Gomes R, Nascimento EFD, Araújo Fábio Carvalho de (2007). [Why do men use health services less than women? Explanations by men with low versus higher education]. Cad Saude Publica.

[28] Novak M, Guest C (1989). Application of a multidimensional caregiver burden inventory. Gerontologist.

[29] Cerentini M (2021). Repercussões na família de baixa renda diante do diagnóstico de Alzheimer na perspectiva do (a) cuidador (a) informal [dissertation]. Universidade Católica de Pernambuco.

[30] Marques MJF, Teixeira HJC, Souza DCDBND (2012). Cuidadoras informais de Portugal: vivências do cuidar de idosos. Trab Educ Saúde.

[31] Chou K, Jiann-Chyun L, Chu H (2002). The reliability and validity of the Chinese version of the caregiver burden inventory. Nurs Res.

